# Effect of ZrO_2_ Nanomaterials on Wettability and Interfacial Characteristics of Al-19Cu-11Si-2Sn Filler Metal for Low Temperature Al to Cu Dissimilar Brazing

**DOI:** 10.3390/nano8100784

**Published:** 2018-10-03

**Authors:** Do-Hyun Jung, Sri Harini Rajendran, Jae-Pil Jung

**Affiliations:** Department of Materials Science and Engineering, University of Seoul, Seoul 02504, Korea; jdh1016@uos.ac.kr (D.-H.J.); harini.phys@gmail.com (S.H.R.)

**Keywords:** ZrO_2_ nanomaterials, wettability, brazing, intermetallic compound, crack propagation

## Abstract

Dissimilar Al 3003 and Cu tubular components were successfully brazed without interface cracking using ZrO_2_ nanomaterials reinforced with Al-19Cu-11Si-2Sn filler. The filler was initially cast using an induction furnace and processed into ring form for brazing. Al-19Cu-11Si-2Sn filler with coarse CuAl_2_ and Si phases (43 and 20 μm) were refined to 8 and 4 μm, respectively, after the addition of 0.1 wt. % ZrO_2_ and shows significant improvement in the mechanical properties. ZrO_2_ nanomaterials’ induced diffusion controlled growth mechanism is found be the responsible for the refinement of CuAl_2_ intermetallic and Si particles. The wettability of Al-19Cu-11Si-2Sn-0.1ZrO_2_ increased to 78.17% on Cu side and 93.19% on the Al side compared from 74.8% and 89.9%, respectively. Increase in the yield strength, ultimate tensile strength, and percentage elongation were noted for the brazed joints. Microstructure of induction brazed joint with 40 kW for 6 seconds using Al-19Cu-11Si-2Sn-0.1ZrO_2_ filler shows thin interfacial CuAl_2_ intermetallic compound along the copper side and inter-diffusion region along the aluminum side and their respective mechanism is discussed. The tensile strength of the joints increased with increasing the nanomaterials addition and shows a base metal fracture. Analysis of fractured samples shows the effectiveness of ZrO_2_ reinforced filler in crack propagation through the filler.

## 1. Introduction

Aluminum and its alloys are often joined to copper to form assemblies that compensate for the increasingly growing demands for engineering applications, including tubes made for refrigeration and heat-exchangers, electrical connectors, foil conductor in transformers, capacitor and condenser foil windings [1]. However, it is a practical challenge to achieve reliable aluminum copper joints due to their distinct physical, chemical, and mechanical properties. Though many investigations have been carried out to achieve reliable joints between aluminum and copper by adopting various joining methods, like cold spraying [2], cladding [3], ultrasound welding [4], vacuum hot pressing [5], roll bonding [6], friction welding, friction stir spot welding (FSSW) [7], friction drilling [8], and brazing [9]. However each method has their own limitations with respect to the geometry of the components, cost, fraction of intermetallic compounds (IMCs), and residual stress concentration [10]. Brazing is a commercial method for joining copper to aluminum tubes in heat exchanger circuits owing to the component geometry and cost purposes [9]. In this case selecting an appropriate filler metal is necessary to provide reliable joints. 

In general, Al4047, which consists of Al-12 wt. % Si, has been used as a filler metal in the brazing industry for heat exchangers because Si increases the fluidity and prevents shrinkage defects during solidification [11]. On the other hand, the formation of large Si-needles in Al-Si alloys impart brittleness to the joint that leads to cracking [11,12]. Recently, Cu is added to reduce a melting point of Al-Si alloy, indeed strong negative enthalpy of mixing between Al and Cu leads to the formation of Al_x_Cu_y_ brittle IMCs, which not only affects the mechanical properties, but also degrades the electrical and thermal conductivity of the brazed joint [13,14]. Moreover, the formation of Al*_x_*Cu*_y_* IMC is more predominant in the joining of copper tubes. Hence, controlling the interface is of prime importance and is always in demand for the heat exchanger applications [15]. 

More recently nanomaterials are considered as a primary candidate for controlling the intermetallic phase during solidification, as well as the interface morphology during joining [16,17]. Nanomaterials are finding a potential scope in Sn-Ag-Cu (SAC) solders as they provide satisfactory results in controlling the Cu_6_Sn_5_ and Cu_3_Sn IMC along the Cu-SAC interface [17]. In the direction of brazing addition of nanomaterials to the filler metal has become an attractive technique of research to suppress the IMC growth and simultaneously refine the Si phase for the significant improvement in brazeability and mechanical properties [18,19,20,21]. Studies on the addition of SiC nanomaterials in Al-Si and Al-Si-Cu alloys have increased the wetting and strength of the joint [22,23].

Though the influence of nanomaterials on the Al based filler metals have been extensively studied in view of wetting properties, mechanical properties, and melting point in the above studies, however, no significant studies have been reported so far on the influence of nanomaterials on the interface during brazing. 

Zirconium dioxide (ZrO_2_) nanomaterials have one of the highest mechanical strength at room temperature among ceramics for engineering. In addition, it is suitable for alloying with various metals because their thermal expansion is close to the metal, and, particularly, they have high resistance against crack propagation in the metal matrix. Furthermore, ZrO_2_ is a potentially attractive ceramic in technological applications and our previous studies shows significant refinement of IMC in ZrO_2_ reinforced Al-Cu-Si alloy [20]. Therefore, in this study, the addition of ZrO_2_ nanomaterials has been investigated to prevent a crack from the brazed joint. In the present work nano ZrO_2_–reinforced Al-19Cu-11Si-2Sn filler has been developed using casting and their advantages over unreinforced Al-19Cu-11Si-2Sn were characterized in terms of interfacial microstructures and mechanical properties.

## 2. Materials and Methods 

### 2.1. Preparation of ZrO_2_ Reinforced Filler Metal

Al-19%Cu-11%Si alloy (compositions are in wt. % unless stated otherwise) was prepared from bulk rods of commercial purity aluminum (99.7%), copper (99.8%), and Al-12Si master alloy. Zirconium dioxide nanomaterials (ZrO_2_, Ditto Technology Co. Ltd., Gyeonggi-do, South Korea, > 99.8% purity) with a mean particle diameter of 50 nm was used as a reinforcement material in the filler metal matrix. For fabrication of Al-19Cu-11Si-2Sn-xZrO_2_ (x = 0.03, 0.05 and 0.1%) nanocomposite, Sn-ZrO_2_ master alloy was fabricated using roll bonding. This procedure was adopted to increase the adhesion of nanomaterials. The surface tension and deformation of Sn can an important role in enhancing the wetting and adhesion of ZrO_2_ nanomaterials. Recently it has been reported that wetting and adhesion are enhanced by the deformation of soft substrates [24].

The required quantity of ZrO_2_ nanomaterials dispersed in ethanol were drop cast on Sn sheets and dried in an oven at 80 °C. The sheets were weighed previously as required for the filler alloy. The sheets are stacked and rolled for a 30% reduction initially. Then, the Sn sheets were sectioned in half and rolled for a 50% reduction. The fabricated Sn-ZrO_2_ sheets were used as master alloy. Al, Cu, and Si of required composition were melted at 750 °C using induction furnace (PSTEK HF-15-TM-CL, Gyeonggi-do, South Korea). The temperature was measured using a K-type thermocouple. The temperature was raised to 900 °C for the addition of Sn-ZrO_2_ foils and held for 20 minutes. A stainless steel impeller was used to disperse ZrO_2_ nanomaterials in the filler metal matrix. The filler alloy with ZrO_2_ nanomaterials was cast in a graphite mold (cooling rate 0.2 °C/min) and then sectioned for microstructure, thermal, and mechanical property analysis. For brazing experiments, the filler alloy was are machined into a ring type filler. Similar experimental procedure was adopted for the fabrication of fillers with different ZrO_2_ additions. 

### 2.2. Dissimilar Al/Cu Brazing

**Figure 1:**
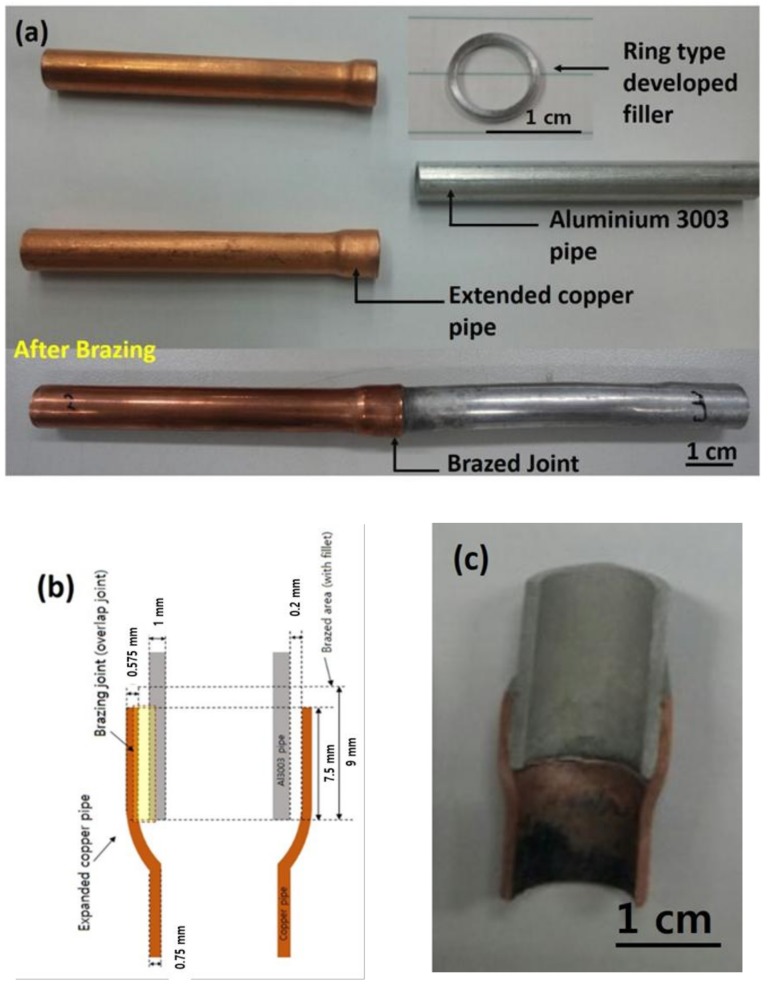
Appearance and schematic diagram for dissimilar Al/Cu brazing using a ring-type filler metal: (**a**) appearance; (**b**) schematic diagram; and (**c**) cross-section of the brazed joint.

Dissimilar Al/Cu brazing experiments were performed with expanded Cu tube (internal diameter of 9.85 mm and external diameter of 11.85 mm), to a non-expanded Al3003 tube (internal diameter of 7.95 mm and external diameter of 9.75 mm) with the ring-type Al-19Cu-11Si-2Sn-ZrO_2_ composite filler metal (see Figure 1a). A non-corrosive brazing flux for Al (Lucas Milhaupt, Inc. DF731, South Pennsylvania Avenue, Cudahy, CA, USA) was used as brazing aid to remove the surface oxides of Cu and Al tubes so that molten fillers can wet and spread up on melting. The induction heating furnace (PSTEK HF-15-TM-CL) at a power input of 30 to 50 kW for 4 to 10 seconds was used for the dissimilar Al/Cu pipe brazing experiments. Figure 1b shows the appearance of the dissimilar Al/Cu tubes brazed samples with Al-19Cu-11Si-2Sn-0.10ZrO_2_ filler metal at power input 40 kW for 8 seconds and Figure 1c represents the corresponding cross-section of the brazing joint where microstructural analysis is carried out in the marked region. 

### 2.3. Characterization of ZrO_2_-reinforced Filler Metal

#### 2.3.1. Microstructure

The cast filler samples were ground metallographically with SiC paper, polished with Al_2_O_3_ suspensions, and etched with a 10 vol. % H_3_PO_4_ solution at 50 °C for 1 minute. The phases present in the microstructure of the filler metal with different ZrO_2_ were examined by analytical scanning electron microscopy (ASEM, JEOL JSM-6010 PLUS/LA, Tokyo, Japan). The phase composition in filler metal were quantified using energy-dispersive spectroscopy (EDS, ASEM, JEOL JSM-6010 PLUS/LA, Tokyo, Japan) equipped with Image-Pro Plus 6.0 program (Media Cybernetics, Inc., Rockville, MD, USA). For EDS compositional analysis, the authors have analyzed approximately 50 spots for each phase and the mean value is considered. High resolution transmission electron microscopy (Philips FEI Technai G2 twin, USA) analysis was used to observe the dispersion of ZrO_2_ nanomaterials in the filler metal matrix.

#### 2.3.2. Brazeability

The spreading test of a filler metal describes the ability of the filler metal to spread over the substrate. Higher spreading ratio indicates better brazeability. High purity Cu (30 × 30 × 0.3 mm) and Al3003 (30 × 30 × 0.3 mm) alloy were used as substrates for the filler spreading test. Five spreading tests were conducted for each filler according to the JIS-Z-3197 [25].

The substrates were ground, polished with SiC paper and cleaned with ethanol to remove any surface contamination. Approximately 0.3 grams of filler metal with a noncorrosive Al flux (Lucas Milhaupt, DF751) was placed on the center of the substrates. The filler metal with substrate was heated to 550 °C in an induction heating furnace and then removed after 30 seconds was allowed to cool in air under room temperature conditions. The spreading ratio was calculated according to the equation below [25]:

(1) $$S=\left(\frac{D-H}{D}\right)X\;100$$

where S is the spreading ratio; H is the height of the filler metal spread; and D is the diameter of the filler metal when it is assumed to be a sphere. In practice, the diameter of the non-uniform spread of the filler metal is measured as follows [26]:

(2) $${\left[D\approx D^{*}=D_{H}^{2}∙D_{V}\right]}^{1/3}$$

where $D^{*}$ is the equivalent diameter of the non-uniform spread; and $D_{H}$ and $D_{V}$ represent the horizontal and vertical diameters of the filler metal spread.

#### 2.3.3. Mechanical Properties

The tensile testing for filler metal was performed according to ASTM E8 standards with crosshead speed of 3 mm/minute using a universal testing machine (UTM MTS 810, Eden Prairie, MN, USA) [27]. For every composition, five samples were tested for reliability of the results. Flat tensile samples were directly casted using a stainless steel mold and machined. From the stress-strain graph, ultimate tensile strength (UTS-maximum value of the tensile stress in the stress–strain graph) and the percentage elongation (ratio of the elongation at the failure to the initial gauge length, multiplied by 100) values are obtained. 

Tensile tests were conducted for the tube in socket brazed Al/Cu joints to determine the effects of ZrO_2_ nanomaterials on the mechanical properties of the joint. Both the ends of Cu and Al pipes were processed flat after brazing, while the center brazed portion is kept as a pipe. Static tensile tests were conducted using conventional methods using a breakage extensometer for determining the displacement in the gauge length. Tensile testing was performed at a crosshead speed of 3 mm/minute using a universal testing machine (UTM MTS 810) as per the ASTM standard E8-M01 and KS B 0801 [27]. The tensile strength and percent elongation were obtained from the stress-strain curves after tensile testing. Five brazed joint samples were tested for each composition. The fracture surfaces were examined using an analytical scanning electron microscope operated at 15 kV.

## 3. Results and Discussion

### 3.1. Analysis of the Filler

#### 3.1.1. Microstructure of ZrO_2_-Reinforced Al-19Cu-11Si-2Sn Filler Metals

**Figure 2:**
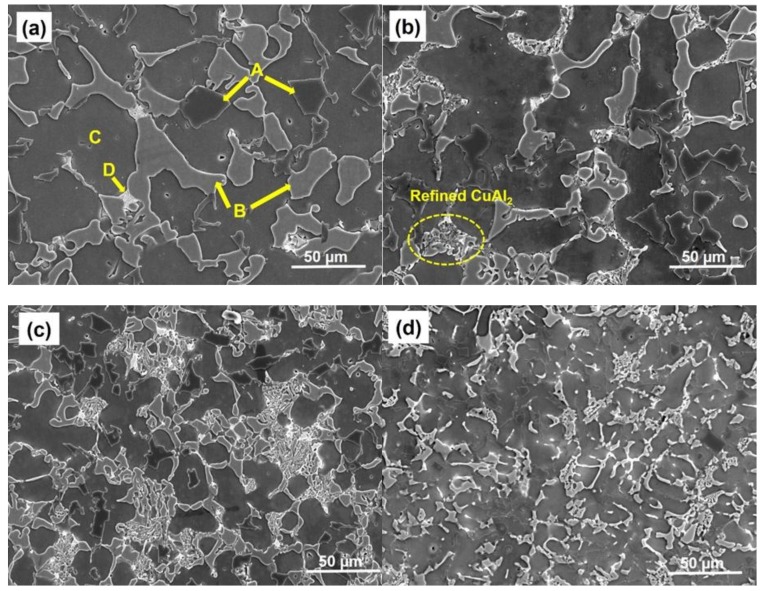
SEM image of nano-ZrO_2_ reinforced Al-19Cu-11Si-2Sn composites with varying addition levels: (**a**) 0% ZrO_2_; (**b**) 0.03% ZrO_2_; (**c**) 0.05% ZrO_2_; and (**d**) 0.10% ZrO_2_.

Figure 2a–d shows the SEM morphology and distribution of phases in as-cast Al-11Si-19Cu-2Sn alloys with the addition of 0.03, 0.05, and 0.1 wt. % of ZrO_2_ nanomaterials, respectively. The EDS results of the point A, B, C, and D marked in Figure 2a were given in Table 1.

Si needles (dark, point A), blocky CuAl_2_ IMC (light gray, point B), Al rich matrix (grey, point C), and Sn rich phase (white, point D) were observed to co-exist in as-cast Al-11Si-19Cu-2Sn alloys in consistent with the previous observations [28,29]. The addition of 0.03% of ZrO_2_ nanomaterials in Figure 2b shows that the blocky CuAl_2_ phases were reduced and refined CuAl_2_ phase start to appear in the microstructure (marked as a dotted circle). In addition, a decrease in Si particle size is also observed. With the increasing addition of ZrO_2_ nanomaterials from 0.03 to 0.05% (Figure 2c), the blocky CuAl_2_ phase and Si particles are further refined. A significant refinement of CuAl_2_ intermetallic phase as well as Si needles in filler alloy is noted for 0.10% ZrO_2_ addition (Figure 2d).

The size of Si particle and CuAl_2_ IMC were calculated at 70 random locations from the SEM microstructure. The addition of ZrO_2_ reduces both Si and CuAl_2_ phase. From the initial size of 43.7 ± 0.9 µm the average size of CuAl_2_ phase has reduced to 38.1 ± 3.4 µm, 19.5 ± 4.0 µm, and 8.58 ± 1.8 µm for the addition of 0.03, 0.05, and 0.1 wt. % of ZrO_2_ respectively. Meanwhile, Si particle size reduced from 19.7 ± 0.4 µm to 11.3 ± 0.9 µm, 7.2 ± 1.2 µm, and 4.2 ± 1.2 µm for the addition of 0.03, 0.05, and 0.1 wt. % of ZrO_2_ respectively. Clearly, the addition of ZrO_2_ nanomaterials has significant control over the formation of Si and Cu IMC phases in Al-11Si-19Cu-2Sn alloy.

The refinement of Al_2_Cu IMC and Si particles in Al-11Si-19Cu-2Sn alloy with ZrO_2_ nanomaterials addition are explained using nanomaterial-induced diffusional growth control approach where the adsorption of nanomaterials on the growing phase during solidification can alter their morphology and size [16,30]. Diffusion controlled grain growth approach using TiC_0.5_N_0.5_ nanomaterials are acknowledged as a novel grain refining method in pure Al [31]. As per the concepts from the model, the presence of ZrO_2_ nanomaterials during solidification of an alloy can contribute to the following possibilities that lead to the refinement of intermetallic phases [31]:

(1) Local change in the temperature of solidification front influencing the growth of α-Al phase.
(2) The ZrO_2_ nanomaterials reduces the diffusion of atoms to the surface of the growing Si and CuAl_2_ phase.
(3) Engulfed ZrO_2_ nanomaterials onto CuAl_2_ or Si phase increases the free energy of their growth by distortion and thus reducing the driving force for growth.
(4) Pinning of the solidification front by ZrO_2_ nanomaterials is explained by the Gibbs-Thomson effect.

**Figure 3:**
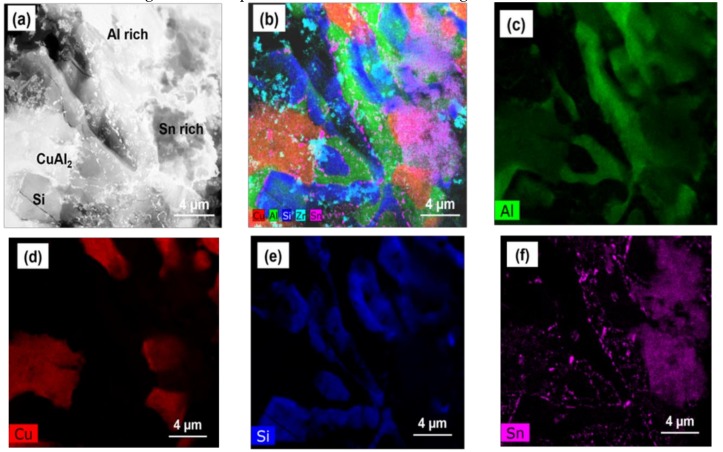

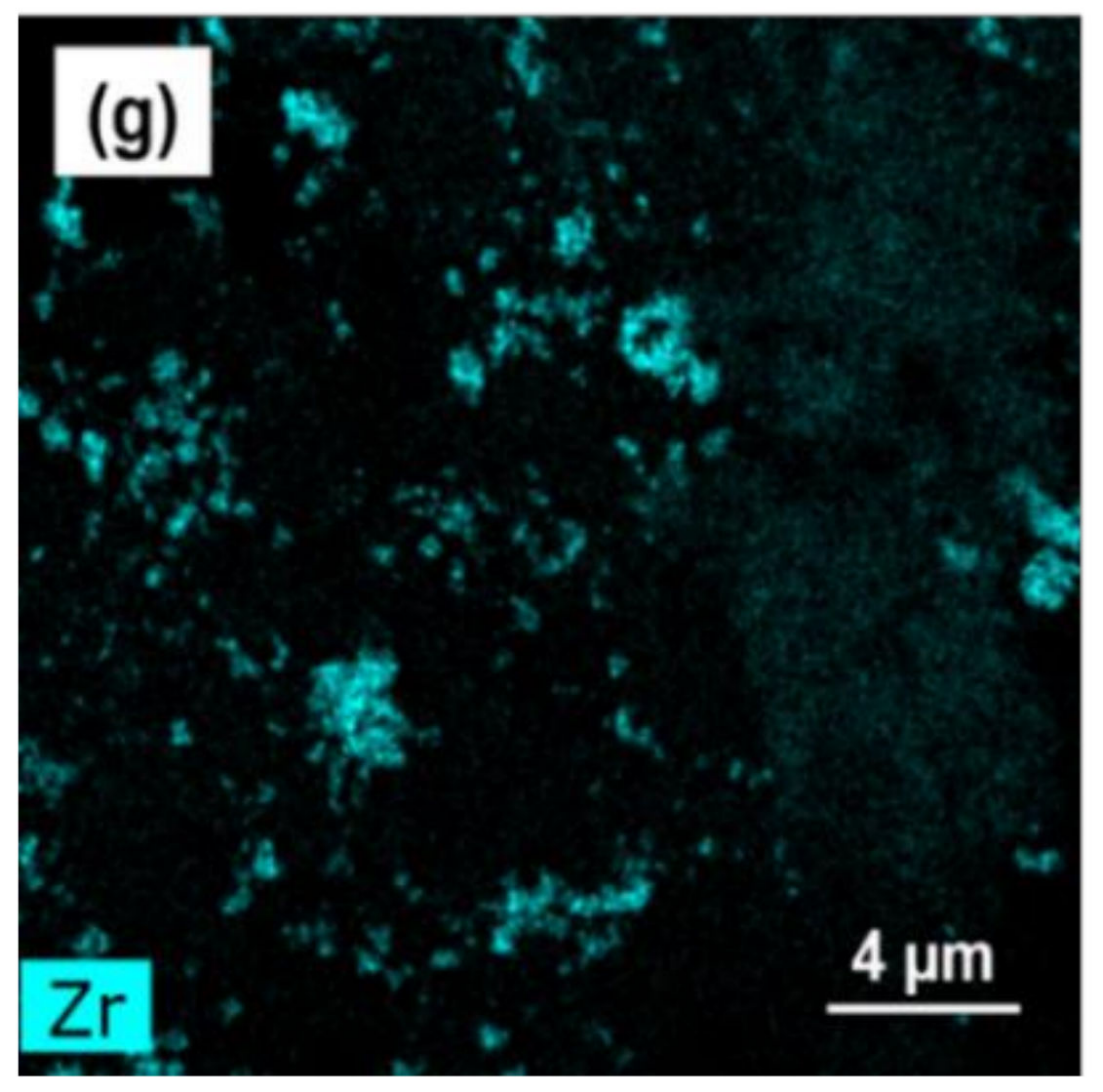
HAADF-STEM images of (**a**) Al-19Cu-11Si-2Sn-0.1% ZrO_2_ nanomaterials; (**b**) corresponding elemental mapping; (**c**–**g**) individual elemental mapping of Al, Cu, Si, Sn, and Zr, respectively.

Figure 3 shows HAADF-STEM images of the Al-19Cu-11Si-2Sn filler with 0.10% ZrO_2_ nanomaterials and the corresponding elemental mapping. Figure 3c–g shows the adsorption of cluster of ZrO_2_ nanomaterials at the growth front of CuAl_2_ phase. From Figure 3b,e adsorption of ZrO_2_ nanomaterials are seen along the Si particles surface. This indicates that ZrO_2_ nanomaterials act as an obstacle to the growth of Si particle and CuAl_2_ IMC during solidification. 

During solidification, the nucleation and growth of primary α-Al dendrites pushes the fine ZrO_2_ nanomaterials continuously ahead of the solid-liquid interface due to a negative Hamaker constant between ZrO_2_ nanomaterials and α-Al. For a nanomaterials–liquid–solid system, the Hamaker constant for system can be given as follows [32]:

(3) $$A_{\mathrm{sys}}=\left(\sqrt{A_{\alpha -\mathrm{Al}}}-\sqrt{A_{L-\mathrm{Al}}}\right)\left(\sqrt{A_{\mathrm{ZrO}2}}-\sqrt{A_{L-\mathrm{Al}}}\right)$$

where $A_{\alpha -\mathrm{Al}}$, $A_{L-\mathrm{Al}}$ and $A_{\mathrm{ZrO}2}$ are the Hamaker constant for α-Al ($A_{\alpha -\mathrm{Al}}$ = 333 ZJ), liquid Al melt ($A_{L-\mathrm{Al}}$ = 266 ZJ), and ZrO_2_ ($A_{\mathrm{ZrO}2}$ = 200 ZJ), respectively [32,33]. For the Al-ZrO_2_ nanocomposite system, the Hamaker constant calculated from Equation 3 is –4.2026 resulting in continuous pushing of the nanomaterials ahead of the solidification front. This increases the concentration of ZrO_2_ nanomaterials in the liquid melt ahead of the solidification front. Figure 2 and Figure 3 confirm the possibility that the nanomaterials-enabled growth control mechanism should be the major contributor for the significant reduction in the size of Si and CuAl_2_ intermetallic phases. 

According to Gibbs-Curie-Wulf law growth of Al_2_Cu IMC is preferred along the crystal plane with the highest attachment energy [34]. Morphology transition in Al_2_Cu can be explained using the oriented attachment mechanism where the distribution of the surface energy affects the final morphology and of crystallite growth [35]. During solidification, initially Al_2_Cu phases nucleate as tiny crystallites. As they grow, single Al_2_Cu crystals orient themselves by reducing the free energy of surface and interface instability, thereby folding to form an L shaped morphology, transforming to an E shaped morphology and, finally, collapse to form a rectangular pattern by the addition of atoms along the preferential crystallographic planes [35]. As seen from Figure 2, with the addition of nanomaterials, the morphology of Al_2_Cu changed from rectangular in Figure 2a to L shaped in Figure 2d. 

Thus, it could be interpreted atomically that, during solidification, the segregation of ZrO_2_ nanomaterials have blocked the diffusion of the Cu atoms to the already nucleated Al_2_Cu phase by getting adsorbed on the preferential growth planes of Al_2_Cu. The regular occurrence of Si particles, i.e., octahedral or twinned shaped through atom adsorption as per the diamond cubic structure [36]. As observed by Wang et al., the regular octahedral or twinned shapes occurs as a direct transition from facetted growth unit along {111} plane evolved from spherical nucleation [37]. Compared with the base Al-Cu-Si-Sn alloy, as shown in Figure 2a, the Si particles morphology in Al-Cu-Si-Sn-0.1ZrO_2_ alloy exhibited a less regular occurrence and irregular Si particle morphology are observed in Figure 2d. 

**Figure 4:**
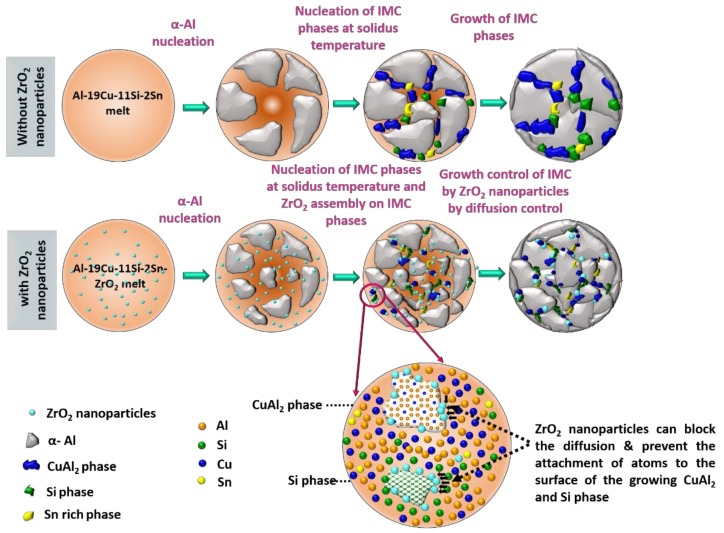
Schematic illustration for Si particle and CuAl_2_ phase growth control by ZrO_2_ nanoparticles in Al-19Cu-11Si-2Sn filler metal.

It was reported that inhibited growth along {111} planes, inhibition growth of planer and vertex directions, and the difference in growth between (111) and (112) directions could contribute to the formation of irregular Si particles [37]. Hence, the morphology change and size reduction observed in ZrO_2_ reinforced fillers could be possibly attributed to the adsorption of ZrO_2_ nanomaterials followed by the inhibition of preferred growing planes of Si. From the above explanations, the schematic of nanomaterials enabled the growth control mechanism for the CuAl_2_ IMC phase and Si particles in nano-reinforced Al-19Cu-11Si-2Sn filler is shown in Figure 4.

#### 3.1.2. Spreading Analysis of ZrO_2_ Reinforced Al-19Cu-11Si-2Sn Filler

**Figure 5:**
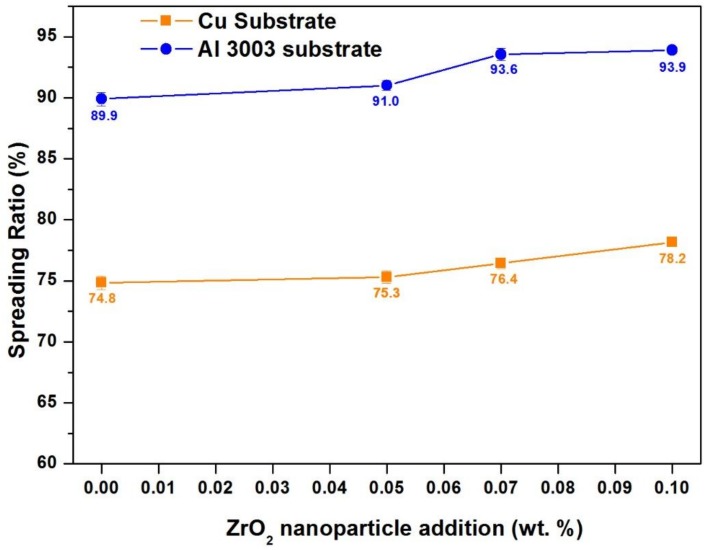
Spreading ratio of Al-19Cu-11Si-2Sn as a function of ZrO_2_ nanomaterials addition.

Spreading of the molten filler has a remarkable influence on the brazing. Figure 5 shows the spreading ratio for Al-19Cu-11Si-2Sn filler alloy on Cu and Al substrates as a function of ZrO_2_ addition. The spreading ratio increases from 74.8% ± 0.5 to 78.2% ± 0.2 for copper and 89.9% ± 0.6 to 93.9% ± 0.4 for Al with the addition of 0.1% of ZrO_2_ nanomaterials. The spreading ratio of the fillers on Al substrate is higher than Cu substrate, due to the mutual solubility of Al-19Cu-11Si-2Sn filler on Al. On Cu substrate, diffusion of Al and Cu forms intermetallic compounds, like Cu_9_Al_4_, Al-Cu, and Al_2_Cu [38].

In nanocomposite solder, it is generally accepted that the segregation of reinforcement particles at the triple point lowers the triple point energy decreasing the wetting angle and increasing the spreading ratio [39]. The increase in the spreading ratio of Al-19Cu-11Si-2Sn filler with increasing ZrO_2_ nanomaterials’ addition can be due to the nanomaterials’ segregation at the triple point. 

#### 3.1.3. Mechanical Properties of ZrO_2_ Reinforced Al-19Cu-11Si-2Sn Filler

Table 2 shows the mechanical properties of ZrO_2_ reinforced Al-19Cu-11Si filler material. All of the standard deviations are being reported with the mean value. Microhardness of Al-19Cu-11Si-2Sn filler alloy increases from 90 to 110 HV with the addition of ZrO_2_ nanomaterials. The increase in the deformation resistance could be attributed by the effective blocking of dislocation motion after the addition of ZrO_2_ nanomaterials. Moreover the continuous pushing of ZrO_2_ nanomaterials ahead of the solid–liquid interface can increase the assembly of ZrO_2_ nanomaterials on to the grain boundary, which can inhibit the deformation during indentation [40].

Comparison of tensile properties of Al-19Cu-11Si-2Sn filler with varying additions of ZrO_2_ nanomaterials shows the apparent increase in the ultimate tensile strength (UTS), yield strength (YS), and % elongation compared with the base Al-19Cu-11Si-2Sn alloy. UTS, YS, and % elongation of 0.1ZrO_2_ reinforced filler were improved by 30%, 25%, and 42%, respectively compared to the base filler alloy. To assess the overall tensile properties, the quality index (Q) was calculated as per the formula given by Drouzy and Richard:

(4) Q=UTS+(150 xlog(% Elongation))

Compared without ZrO_2_ nanomaterials, filler with 0.1% ZrO_2_ nanomaterials shows a 38% increase in the quality index. The increase in mechanical properties can be due to the refinement of Si particles and CuAl_2_ IMC, as well as the presence of ZrO_2_ nanomaterials in the matrix. 

As per the investigations carried on continuum and micromechanical models to understand the strengthening mechanisms of composites, the major mechanisms that contributes to the increase in tensile strength of the filler metal by the ZrO_2_ nanomaterials was attributed to the combined effects [41]:

(5) $$\sigma_{y}=\sigma_{y}^{\mathrm{LT}}+\sigma_{y}^{\mathrm{CTE}}+\sigma_{y}^{\mathrm{Oro}}+\sigma_{y}^{\mathrm{GND}}$$

where $\sigma_{y}^{\mathrm{LT}}$ is the strengthening contribution by load transfer through shear stress along the interface between the components. Apart from ZrO_2_ reinforcement, as CuAl_2_ intermetallic compounds and Si particles also share the interface with the Al matrix. The morphology and size of CuAl_2_, as well as Si particles in Al-19Cu-11Si-2Sn alloy accounts for the strengthening through load transfer. For ZrO_2_ nanomaterials, the contribution of the load transfer effect is calculated as follows [41]:

(6) $$\Delta \sigma_{\mathrm{load}}=0.5V_{p}\sigma_{\mathrm{um}}$$

where $V_{p}$ is the volume fraction of ZrO_2_ nanomaterials and $\sigma_{\mathrm{um}}$ is the yield strength of the unreinforced matrix. $\sigma_{y}^{\mathrm{CTE}}$ is the strengthening contribution due to the generation and pile up of dislocations along the particle-matrix interface because of the difference in thermal contraction between the matrix and reinforcement during solidification. Enhanced dislocation density due to the mismatch in the CTE between the filler metal matrix and ZrO_2_ nanomaterials is as follows:

(7) $$\Delta \sigma_{y}^{\mathrm{CTE}}=\eta \mathrm{Gb}\sqrt{\frac{12V_{p}\Delta \alpha \Delta T}{\mathrm{bd}\left(1-V_{p}\right)}}$$

where η is a constant, b is the Burgers vector, G is shear modulus, Δα is CTE difference between the matrix and nanomaterials, ΔT is the difference in the processing temperatures, and d is the particle size. $\Delta \sigma_{y}^{\mathrm{oro}}$ is the strengthening contribution due to the resistance of ZrO_2_ nanomaterials to passage of dislocations as calculated by Ashby-Orowan equation as follow [42]:

(8) $$\Delta \sigma_{y}^{\mathrm{oro}}=\frac{2\mathrm{mGb}\;\ln\left\{d|2b\right\}}{\left[\left(1.18\right)4\pi \left(I_{p}-d\right)\right]}$$

where m is Tayler factor and $I_{p}$ is the inter-particle separation. Geometrically necessary dislocations are generated along the matrix and ZrO_2_ nanomaterials interface area due to their elastic modulus and yield strength differences as a course of tensile load.$\Delta \sigma_{y}^{\mathrm{Geo}}$ is the strengthening contribution by geometrically-necessary dislocations as given by [43]:

(9) $$\Delta \sigma_{y}^{\mathrm{Geo}}=\beta G\sqrt{\frac{V_{p}\epsilon b}{d}}$$

where β is geometric factor and ϵ is the matrix plastic strain.

### 3.2. Aluminum to Copper Dissimilar Brazing

To understand the effect of ZrO_2_ nanomaterials induction brazing is carried with nano ZrO_2_ Al-19Cu-11Si-2Sn filler. After brazing, the joints were analyzed based on the filler metal melting, penetration and dissolution. Filler metal begins to melt, flows readily, and fills the tiny space between the sockets. During brazing, dissolution of filler metal into the base metal is unavoidable and intensifies with prolonged brazing time and higher brazing temperature. Dissolution of filler metal alters the chemical composition of base metal, decrease its melting temperature, thus, damaging the base metal. The condition with the complete melting and penetration of filler metal ring into sockets without damaging the Al pipe is considered as good bonding. 

**Figure 6:**
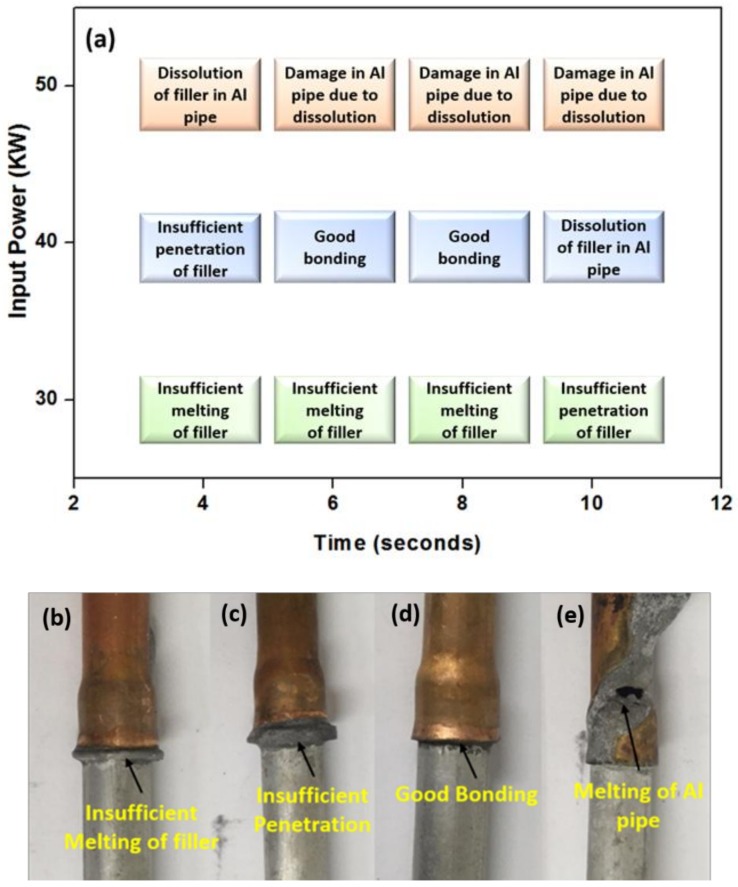
(**a**) Investigation of nature of the joint as a function of input power and brazing time; and their appearances (**b**) insufficient melting of filler, (**c**) insufficient penetration, (**d**) good bonding, and (**e**) melting of Al pipe.

Figure 6 enlists the nature of the brazed samples obtained with respect to the power input and brazing time. The ring-type 0.1% ZrO_2_ reinforced Al-19Cu-11Si-2Sn filler metal with a diameter of 10 mm and thickness of 1.5 mm was used as the brazing filler alloy. In the case of a power input of 30 kW, the melting and penetration of the filler metal were insufficient at brazing times between 4 and 10 seconds. Whereas, an input power of 40 kW shows good bonding for 6 and 8 seconds. Meanwhile, increasing the input power further to 50 kW damages the Al pipe by melting and dissolution. Figure 6b–e shows the nature of the joints discussed above. Figure 6b shows the condition where the filler metal was not melted whereas Figure 6c shows the insufficient penetration where the filler metal was partially melted. Figure 6d shows the nature of good joint. It shows a bright, shiny, and uniform brazing appearance where the brazed joint become a flat and hollow curved fillet by the capillary phenomenon of filler metal. This means that more filling of liquid filler metal is observed inside the Al and Cu pipes. 

On the other hand, damaged brazing joint as shown in Figure 6e appears with an excessive dissolution and melting of an Al pipe. The increase in brazing time and power input causes a higher heat energy and heat input [44].

Therefore, wetting and excessive melting of both the filler and base metal occur, resulting in the severe damage to the brazed joint at a higher brazing time and power input. Dissolution of the base metal by the molten filler metal is related to the saturation solubility of the base metal in the filler metal as well as the diffusion velocity of the filler metal, which can be explained by the Nerst-Brunner equation below [45,46]:

(10) $$C=C_{S}\left[1-\exp\left(-K\frac{S}{V}t\right)\right]$$

where C is the concentration of the base metal dissolved in the molten filler metal; $C_{S}$ is the saturation solubility; K is the dissolution rate constant; V is the volume of the molten filler; S is the solid-liquid interface area between the molten filler metal and the base metal; and t is the brazing time. Equation (10) can also be expressed with a differential equation as follows:

(11) $$\frac{\mathrm{dC}}{\mathrm{dt}}=K\frac{S}{V}\left(C_{S}-C\right)$$

Assuming that the amount of solute that can dissolve into the molten filler metal is equal, the dissolution parameter P can be expressed as:

(12) $$P=\mathrm{Kt}=h\left[\frac{\ln\left(X_{0}X_{t}+\rho h\right)}{\rho h\left(X_{0}-X_{t}\right)}\right]$$

where $X_{t}$ is the width of the dissolved base metal at time t; $X_{0}$ is the width of the saturation solubility; ρ is the density of the molten filler metal for the density of the base metal; and h is the half of the width of the initial molten filler metal. In a typical brazed joint, the saturation solubility has a great effect on the dissolution of the base metal because of the difference in composition between the base metal and filler metal.

### 3.3. Analysis of the Al/Cu Brazed Joint

#### 3.3.1. Interface Analysis 

**Figure 7:**
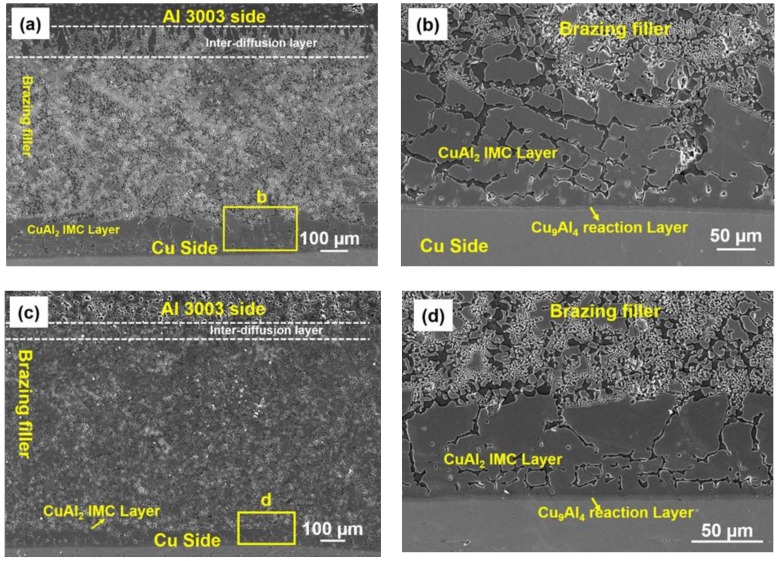
Al3003/Al-19Cu-11Si-2Sn/Cu interface analysis in brazed samples (**a**) and (**b**) without ZrO_2_ nanomaterials and (**c**) and (**d**) with 0.1% ZrO_2_-reinforced filler metal.

Figure 7a–c shows the cross-sectional microstructure of the Cu/Al joint induction brazed at 40 KW for 6 seconds using Al-19Cu-11Si-2Sn and Al-19Cu-11Si-2Sn-0.1ZrO_2_ nanocomposite filler, respectively. The interface of both joints exhibit good bonding without any defects. In the Al/filler interface, during melting of the filler, the solubility of the filler in Al3003 creates an inter diffusion zone. During solidification, the liquid phase on inter-diffusion zone crystallized on the solid Al3003 base metal with vertical columnar crystals. 

In the Cu side, Al and Cu forms a series of intermetallic compounds. The predominant IMC along the Al/Cu interface for Cu clad Al alloys are Cu_9_Al_4_, AlCu, and Al_2_Cu_3_. The SEM analysis along the filler/Cu interface, as shown in Figure 7b,d, shows the formation of continuous intermetallic layer. This can be attributed to Cu_9_Al_4_ IMC and the immediate layers are Al_2_Cu. Compared with the interface brazed using Al-19Cu-11Si-2Sn, the filler with the 0.1% of ZrO_2_ nanomaterials show a relatively thin inter-diffusion layer along the Al/filler and thin Al_2_Cu IMC along the filler/Cu interface. 

**Figure 8:**
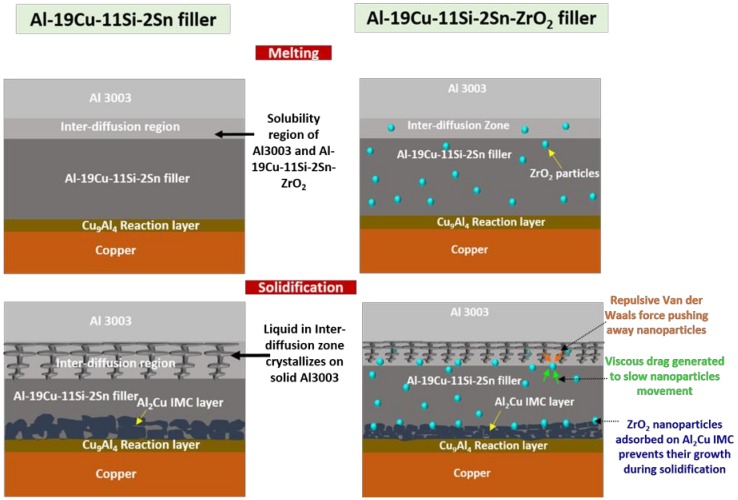
Schematic illustration for ZrO_2_ nanomaterials in Al/filler and Cu/filler interface in Al-19Cu-11Si-2Sn filler metal.

The presence of nanomaterials in the filler has significant influence on the dendrite growth on the Al3003 side. During solidification of the filler, low Hamaker constant of ZrO_2_ nanomaterials with respect to Al melt creates a repulsive van der Waals force pushing away the nanomaterials from the advancing solidification front. The repulsive Van der Waals force is given as follows [33]:

(13) $$F_{\mathrm{RVF}}=-\frac{A_{\mathrm{sys}}R}{6D^{2}}$$

where R is the radius of nanomaterials, D is gap between solidification front and nanomaterials and $A_{\mathrm{sys}}$ is Hamakar constant of system calculated using Equation (3). Corresponding to repulsive force, viscous drag force will be generated in the melt to slow down the moving nanomaterials. The viscous drag force is given by [33]:

(14) $$F_{\mathrm{vis}}=6\pi \left(\frac{D}{D-2D_{s}}\right)\nu \eta \frac{R^{2}}{D}$$

where $D_{s}$ diameter of liquid molecule, ν is the velocity of the nanomaterials relative to the liquid and η is viscosity of bulk liquid. This viscous drag force might also slow down the velocity of diffusing atoms from the liquid melt towards the Al substrate, thus reducing the inter-diffusion layer thickness as explained in the schematic in Figure 8. For the Cu substrate, ZrO_2_ nanomaterials effectively reduced the thickness of Al_2_Cu IMC. The adsorption of surface active ZrO_2_ nanomaterials on the CuAl_2_ growth planes can affect IMC growth in consistent with the theory of the absorption of surface-active materials as shown in the schematic in Figure 8 [47].

#### 3.3.2. Tensile Properties of the Brazed Joint

**Figure 9:**
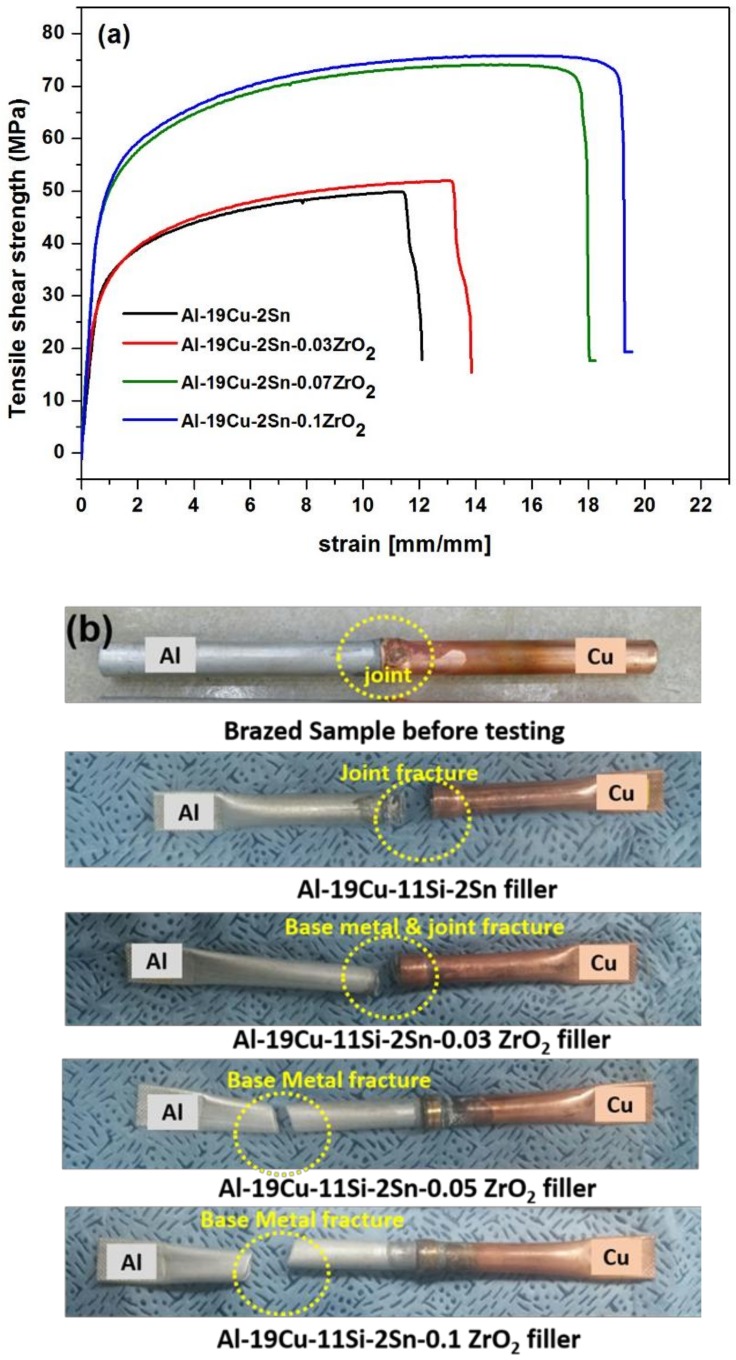
(**a**) Tensile stress strain curve of the brazed joint with respect to increasing nanomaterials addition and (**b**) the fractured samples after the tensile test.

The tensile strength of Al/Cu brazed joint using Al-19Cu-11Si-2Sn filler and ZrO_2_ nano-reinforced Al-19Cu-11Si-2Sn filler is shown in Figure 9a. The tensile shear strength of the Al/Cu joints induction brazed with Al-19Cu-11Si-2Sn filler is 45.1 MPa. Addition of 0.03, 0.05, and 0.1% of ZrO_2_ nanomaterials in Al-19Cu-11Si-2Sn filler increased the tensile strength to 46.4, 48.6, and 50.5 MPa, respectively. The desired fracture for the good brazed joint is the fracture in the base metal rather than the fracture in the joint. Figure 9b shows the corresponding fractured samples with encircled fracture area. Joint fracture was observed in Al-19Cu-11Si-2Sn filler whereas Al-19Cu-11Si-2Sn filler with 0.03ZrO_2_ nanomaterials show the combination of joint and base metal fracture. With increasing ZrO_2_ nanomaterials addition to 0.05 and 0.1%, fracture occurred on Al3003, thus confirming a sound brazed joint. The formation of brittle Al_2_Cu at Cu/filler interface initiates the cracks to the brazing joint. The base metal fracture observed with the addition to 0.05 and 0.1% ZrO_2_ nanomaterials can be attributed to the refinement of CuAl_2_ IMC along the interface, as well as a thin inter-diffusion layer on Al3003.

#### 3.3.3. Fracture Analysis of the Brazed Joint

**Figure 10:**
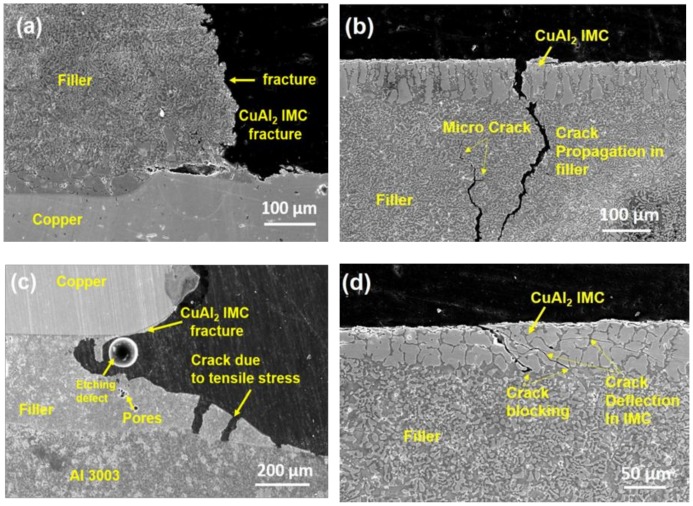
Fracture analysis of the brazed joint (**a**) and (**b**) using Al-19Cu-11Si-2Sn filler and (**c**) and (**d**) using Al-19Cu-11Si-2Sn-0.03 ZrO_2_ nanomaterials reinforced filler.

Joint fracture occurred in samples brazed using Al-19Cu-11Si-2Sn and Al-19Cu-11Si-2Sn-0.03ZrO_2_ fillers. Cross-sectional fracture analysis were carried out at the copper edge and on the aluminum edge, as shown in Figure 10a to 10d for samples brazed using Al-19Cu-11Si-2Sn and Al-19Cu-11Si-2Sn-0.03ZrO_2_ fillers. Figure 10a and 10b shows the fracture on the Cu and Al edge for Al-19Cu-11Si-2Sn filler. It can observed that the fracture has occurred in filler metal, as well as the intermetallic layer. It is well observed that crack propagation along the CuAl_2_ IMC on the copper side and propagated through the filler alloy with few micro crack formation, thus leading to the fracture along the filler as observed in Figure 10a. Similarly, Figure 10c shows the cross-sectional fracture analysis carried at the copper edge in sample brazed using Al-19Cu-11Si-2Sn-0.03ZrO_2_ filler. 

Since the fracture mode is a combination of filler and base metal fracture, part of the Al base metal is also observed along with copper. It can be seen that fracture originated along the CuAl_2_ IMC on the Cu side while on the Al side only tensile cracks are observed. No cracks are observed on the Al3003/filler interface. Figure 10d shows the fractured part along the aluminum edge sample brazed using Al-19Cu-11Si-2Sn-0.03ZrO_2_ filler. Similar to Figure 10b, crack propagation is observed throughout the CuAl_2_ top surface, as well as through the CuAl_2_ IMC. Unlike Figure 10b, crack propagation is not through the filler, rather the crack is deflected at the CuAl_2_ filler interface. In addition, CuAl_2_ IMC particle cracking is seen due to micro cracks branching from the main crack.

Crack propagation occurs as a mutual competition between intrinsic damage mechanisms encouraging the crack propagation ahead of the tip and extrinsic crack-tip shielding mechanism retarding crack growth [48]. In metals, micro cracks or voids in the highly stressed region are the intrinsic mechanism which contributes to crack growth by intergranular cracking or micro void coalescence [49]. Though micro crack formation, crack bridging, etc., are the extrinsic mechanism for crack tip shielding in ductile materials, in the presence of second phase particles, crack deflection is the prominent extrinsic mechanism responsible for crack tip shielding [50].

Crack propagation selection depends on the path of low microstructural resistance. In Al-19Cu-11Si-2Sn filler the crack initiated along the Al_2_Cu IMC during tensile testing propagated through the filler metal along with few micro cracks which eventually lead to fracture, whereas in Al-19Cu-11Si-2Sn-ZrO_2_ filler, cracks are blocked and deflected along the CuAl_2_/filler interface. Thus, confirming that ZrO_2_ nanomaterials adsorbed on CuAl_2_ IMC provide high microstructural resistance for crack propagation. ZrO_2_ nanomaterials along the interface might block the dislocations generated from the advancing crack tip resulting in crack branching along the CuAl_2_ intermetallic particles.

The study suggests that nanoparticle addition can be effectively utilized to improve the mechanical strength in brazing fillers as well as to tailor their interface properties, thus producing a sound joint. 

## 4. Conclusions

In this work, aluminum 3003 and copper cylindrical tubes were joined through induction brazing. Ring-type ZrO_2_ nanomaterial-reinforced Al-19Cu-11Si-2Sn filler metal was employed at 30, 40, and 50 kW input power for varying times ranging from 4, 6, 8, and 10 seconds. Filler metal analysis, interface analysis after brazing, and tensile testing of the brazed joints were carried out to understand the joint quality. The observations are summarized as follows:

(1) SEM analysis shows a significant size reduction in Si particles and CuAl_2_ IMC with the addition of ZrO_2_ nanomaterials. This phenomenon was explained using ZrO_2_ nanomaterial-induced phase growth control during solidification of the Al-19Cu-11Si-2Sn filler alloy. TEM confirms the presence of ZrO_2_ nanomaterials along the surface of Si particles and CuAl_2_ IMC justifying phase growth control of nanomaterials.
(2) Addition of ZrO_2_ nanomaterials increases the spreading ratio of filler on Cu and Al substrates. Furthermore, ZrO_2_ nanomaterials added to Al-19Cu-11Si-2Sn filler showed better mechanical properties in terms of microhardness and tensile properties.
(3) The joints with varying input power and brazing time demonstrated a good bonding for 40 kW input power for 6 and 8 seconds.
(4) Interface analysis of joint brazed at 40 kW, 8 seconds with Al-19Cu-11Si-2Sn and Al-19Cu-11Si-2Sn-ZrO_2_ demonstrated the formation of continuous Cu_9_Al_4_ reaction layer and discontinuous Al_2_Cu IMC on the Cu side, and formation of an inter-diffusion layer on the Al3003 side. However, a thin IMC and inter-diffusion layer is formed using Al-19Cu-11Si-2Sn-ZrO_2_ filler. ZrO_2_ nanomaterials in the filler are responsible for interface modification by surface adsorption phenomenon.
(5) The tensile test results of the brazed joint justified the importance of ZrO_2_ nano-reinforced Al-19Cu-11Si-2Sn filler to join Al and Cu joints. Tensile strength increased linearly with the concentration of ZrO_2_ nanomaterials in filler alloy. Joints with 0.05 and 0.1 wt. % ZrO_2_ added filler shows base metal fracture confirming sound joint.
(6) Fracture analysis of the brazed joint shows that ZrO_2_ nanomaterials adsorbed on Al_2_Cu IMC block the crack propagation along the interface which leads to crack branching. In filler without ZrO_2_ nanomaterials, cracks propagated through the filler metal leading to fracture.

## Figures and Tables

**Table 1:** Compositional analysis of various phases in Al-19Cu-11Si-2Sn filler from Figure 2.

Mark	Composition (at. %)				Phase
	Al	Si	Cu	Sn	
A	-	100	-	-	Si phase
B	65.3	-	34.7	-	CuAl_2_ IMC
C	92.3	1.4	6.0	0.3	Al rich phase
D	2.5	0.4	3.2	93.9	Sn rich phase

**Table 2:** Microhardness and tensile properties of filler metal with various ZrO_2_ concentration (reported individual values are the average of 5 samples tested).

S.N0	Samples	Micro Hardness (HV)	Tensile Properties			Quality Index(MPa)
			Yield Strength (MPa)	Ultimate Tensile Strength (MPa)	% Elongation	
1	Al-19Cu-11Si-2Sn	89.4 ± 1.2	98.2 ± 1.1	124.7 ± 2.3	1.9 ± 0.2	167.2
2	Al-19Cu-11Si-2Sn-0.03ZrO_2_	93.4 ± 0.9	103.4 ± 2.1	132.4 ± 1.1	2.2 ± 0.3	182.9
3	Al-19Cu-11Si-2Sn-0.05ZrO_2_	107.8 ± 1.3	117.3 ± 1.7	157.4 ± 4.1	2.9 ± 0.1	225.6
4	Al-19Cu-11Si-2Sn-0.1ZrO_2_	108.1 ± 1.4	123.4 ± 1.4	162.3 ± 3.3	2.9 ± 0.2	231.4
